# Hybridization between two recently diverged Neotropical passerines: The Pearly-bellied Seedeater *Sporophila pileata*, and the Copper Seedeater *S*. *bouvreuil* (Aves, Passeriformes, Thraupidae)

**DOI:** 10.1371/journal.pone.0229714

**Published:** 2020-03-27

**Authors:** Cesar A. B. Medolago, Mariellen C. Costa, Luis F. Silveira, Mercival R Francisco

**Affiliations:** 1 Programa de Pós Graduação em Ecologia e Recursos Naturais, Universidade Federal de São Carlos, São Carlos, São Paulo, Brazil; 2 Seção de Aves, Museu de Zoologia da Universidade de São Paulo, São Paulo, São Paulo, Brazil; 3 Departamento de Ciências Ambientais, Universidade Federal de São Carlos, Sorocaba, São Paulo, Brazil; National Cheng Kung University, TAIWAN

## Abstract

The small Neotropical finches called capuchinos are outstanding because they have experienced one of the most recent and explosive avian radiations ever documented for birds. Despite very low morphological and niche divergence among species, many of them are reproductively isolated when in sympatry due to strong sexual selection in plumage traits. However, a specific pair of mostly parapatric species, the Pearly-bellied, *Sporophila pileata*, and the Copper Seedeaters, *S*. *bouvreuil*, has confounded taxonomists because individuals with intermediate color patterns can be found. By analyzing diagnostic COI mtDNA sequences and adult male plumage we provide evidence for hybridization. Paternity tests using microsatellites also indicated that representatives with intermediate plumage pattern can be fertile. Our findings are consistent with the classification of *S*. *bouvreuil* and *S*. *pileata* as distinct taxa, but we demonstrate that the sexual selection mechanisms involved in the isolation of other reproductively sympatric capuchinos are not applicable to this pair of species, likely because of reduced barriers to mate recognition.

## Introduction

Recently diverged taxa can be poorly distinguishable with genetic markers due to hybridization and genetic introgression after secondary contact, or to incomplete lineage sorting [1, 2]. As a result, discordances between genomic evolutionary history and phenotypes often occur, making taxonomic resolution a challenging task [1, 3, 4]. The group of small Neotropical finches named ''capuchinos'' is one such example. These birds form a monophyletic group of 12 species within the genus *Sporophila* (Aves, Thraupidae), and like the other congeners, they are sexually dimorphic and are characterized by the specialization in the consumption of grass seeds [5–7]. The capuchinos are distributed from northern South America to most of Brazil, eastern Bolivia, northeastern Argentina, and Uruguay [5]. They are smaller (10 cm) than the other *Sporophila* and are very homogeneous in shape, size, and also in behavioral aspects [5, 6, 8, 9]. Males of the different species can be diagnosed by their rich nuptial plumage patterns, with tonalities varying mostly from cinnamon to chestnut, combined with black, white, and gray parts, but the females and males in eclipse plumage are predominantly brown to ochraceous, which make them poorly distinguishable by morphology [5, 10]. Eight of the species: *S*. *pileata*, *S*. *cinnamomea*, *S*. *hypochroma*, *S*. *hypoxantha*, *S*. *melanogaster*, *S*. *nigrorufa*, *S*. *palustris*, and *S*. *ruficollis* (and certainly also the recently described *S*. *iberaiensis*) exhibit very low divergence even with the use of thousands of molecular markers and show no reciprocal monophyly, which is attributed to a very recent and rapid radiation occurred during the Pleistocene [10–13]. As these species occur mostly southern from the Amazon basin, they are called southern capuchinos [11, 12, 14]. Exceptions are *S*. *bouvreuil*, which is the sister taxa of this subgroup, and the Amazonian *S*. *minuta* and *S*. *castaneiventris* [13]. These birds are extraordinary because most species not only reproduce in sympatry in floodable areas from southern Brazil and Argentine, but they also migrate and forage together in northernmost wintering areas [6, 9, 15, pers. obs.].

Despite the recent diversification, the unresolved polytomy, and very low niche divergence, the classification of the southern capuchinos (*S*. *cinnamomea*, *S*. *hypochroma*, *S*. *hypoxantha*, *S*. *melanogaster*, *S*. *nigrorufa*, *S*. *bouvreuil*, *S*. *pileata*, *S*. *palustris*, *S*. *ruficollis*, and *S*. *iberaiensis*) as distinct species has been unquestionable. Although there is evidence that song recognition can help to maintain reproductive isolation between at least one pair of species of southern capuchinos in breeding areas (*S*. *palustris* and *S*. *hypoxantha*) [16], an elegant genome-wide study [10] demonstrated that the maintenance of distinct male phenotypes in sympatry, i.e. *S*. *hypoxantha*, *S*. *melanogaster*, *S*. *pileata*, *S*. *nigrorufa* and *S*.*palustris* is the result of strong and rapid selection in sexually selected plumage traits [10]. The existence of these species as reproductively isolated entities is also evidenced by the lack of males with intermediate plumage patterns in the wild and in museum skins [17, pers. obs.], which would often be the first line of evidence for hybridization [18, 19]. Although some color pattern variations have been described for southern capuchino species, i.e. the “uruguaya” morph for *S*. *hypoxantha*; the “xumanxu” morph for *S*. *melanogaster*, and the “caraguata” morph for *S*. *ruficollis* (revised in [17]), they are very rare in nature and it is unclear if they have derived from hybridization [17, pers. obs.]. Yet, there has been no investigations on genetic evidence for hybridization in this group.

Among the southern capuchinos, *S*. *bouvreuil* is the only species that can be clearly distinguishable from the others by molecular markers [13] and has a mostly divergent geographic distribution, that ranges from northern South America, down to western Minas Gerais and eastern São Paulo state, Brazil, where its distribution overlaps with the northern populations of *S*. *pileata* [see 20, 21]. Here we combined mtDNA haplotypes, male nuptial plumage patterns, and offspring paternity tests using microsatellites to demonstrate the occurrence of hybridization between *S*. *bouvreuil* and *S*. *pileata* in their contact zone, explaining the origin of individuals with intermediate plumage pattern that have long confounded taxonomists [20, 21]. Capuchinos are one of the most recent and explosive avian radiation ever documented, and our findings also provide new insights into the mechanisms involved in the diversification of this group.

## Materials and methods

### Study species

The Pearly-bellied (*S*. *pileata*) and the Copper (*S*. *bouvreuil*) seedeaters are indistinguishable by morphometric analyses but they can be clearly diagnosed based on their color patterns [21]. They share similar wing, tail, and pileum colorations, but the Copper Seedeater is characterized by reddish-brown ventral and dorsal regions, while *S*. *pileata* presents light-gray ventral plumage and streaked dark-gray brown upperparts. Notably, a number of individuals described in the literature for central São Paulo state presented an intermediate plumage patterns, and were so far considered as variants of *S*. *pileata* [21]. Individuals with the typical *S*. *pileata* pattern can be found in southern and central Brazil in the states of Rio Grande do Sul, Santa Catarina, Paraná, São Paulo, Mato Grosso do Sul, Minas Gerais and Goiás, and in adjacent countries (Paraguay and Argentina). *Sporophila bouvreuil* is distributed from Suriname and French Guiana to central and northeastern Brazil, down to western Minas Gerais and eastern São Paulo state [20, 21]. Their nests are small deep cups (~6 cm in diameter), built mainly of grass stems, placed in herbaceous vegetation of open floodable areas, 15–70 cm above ground [22]. Clutch sizes consist of two eggs (rarely three), and like all of the *Sporophila*, they are socially monogamous and males are highly territorial during the breeding season [16, 22].

### Study areas

**Fig 1:**
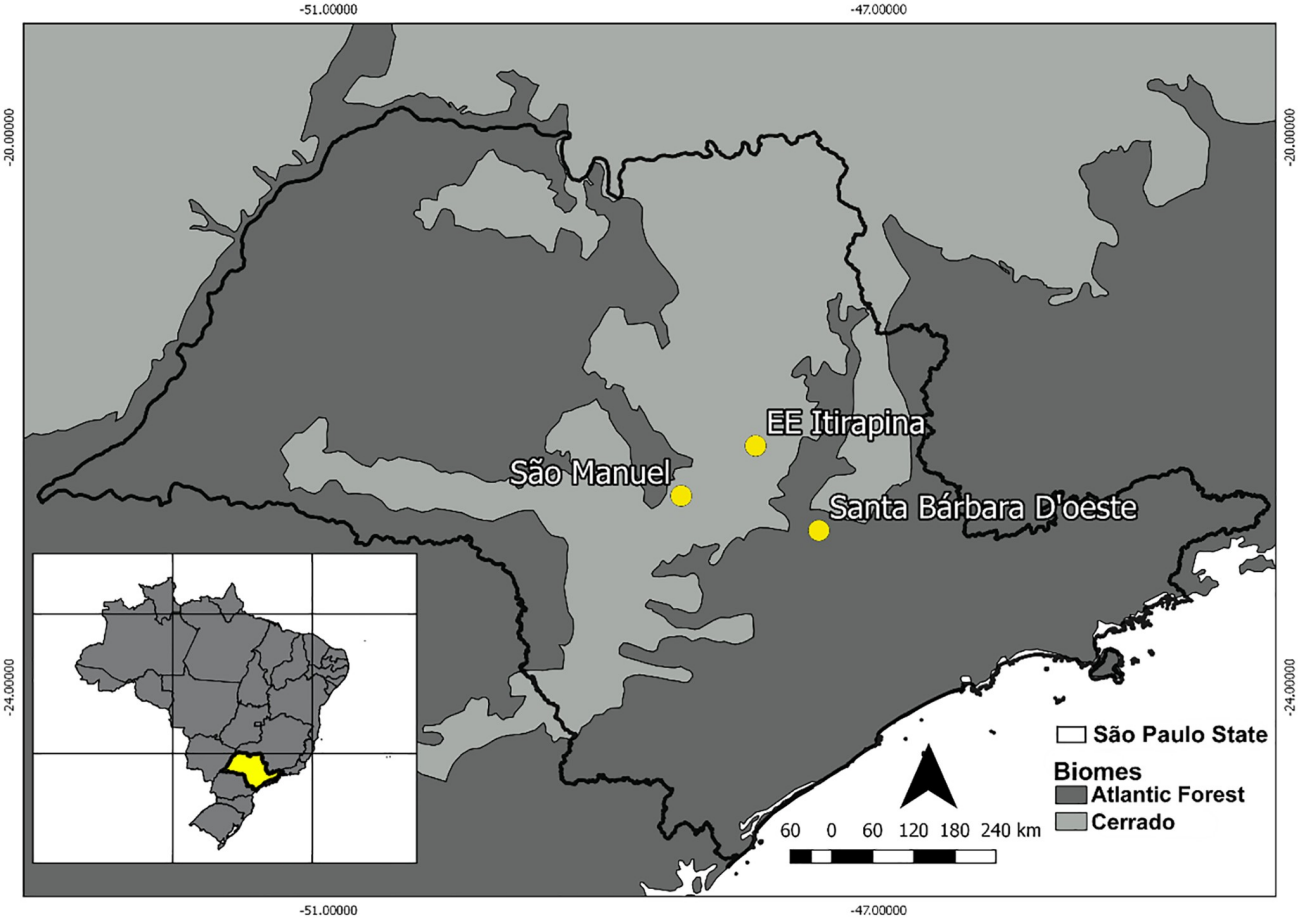
Study areas in central São Paulo state, Southeastern Brazil. Santa Bárbara d’Oeste (SBO), São Manuel (SM), and Estação Ecológica de Itirapina (EEI).

We sampled birds in three areas from Central São Paulo state, southeastern Brazil: Santa Bárbara d’Oeste (SBO) (22°51’S, 47°26’W), Estação Ecológica de Itirapina (EEI) (22°14’S, 47°53’W), and São Manuel (SM) (22°36’S, 48°26’W) (Fig 1). SBO is a complex of three perennial lakes, with approximately 6, 14, and 45 ha of water surface, located in a privet farm from the municipality of Santa Bárbara d’Oeste. These lakes are only about 0.3 to 1 m in water depth, which permits the growing of abundant emergent vegetation, especially representatives of the genera *Rhynchospora* and *Cyperus*. Further, their edges are surrounded by a 50 to 150 m belt of open humid areas where the grass *Andropogon bicornis* and other herbaceous vegetation predominate. These lakes are 300 to 800 m apart from each other and they are embedded in a matrix of sugar cane plantations. EEI is a 2,300 ha conservation unit located in the municipalities of Itirapina and Brotas. It holds one of the last well-preserved areas of the Cerrado ecosystem from São Paulo state, with phytophysiognomies varying from open, floodable or non-floodable fields, to natural forest patches [23]. In SM, birds were sampled in a mosaic of herbaceous marshlands and anthropic grasslands, across a 1.2 km transect established in the edges of Tietê river, in the municipality of São Manuel. In these areas, capuchinos are present from mid-October to March, with reproductive activities concentrated from December to early February (unpublished data). The climate in the region is mesothermic, with a hot and rainy season from October to March (average temperature of 22.8°C, and precipitation of ~1,100mm), and a dry and cold season from April to September (average temperature of 18.8°C, and precipitation of 300 mm [24]. These areas are 70 (IT to SM) to 100 km (SBO to SM) apart.

### Field work and DNA sampling

**Fig 2:**
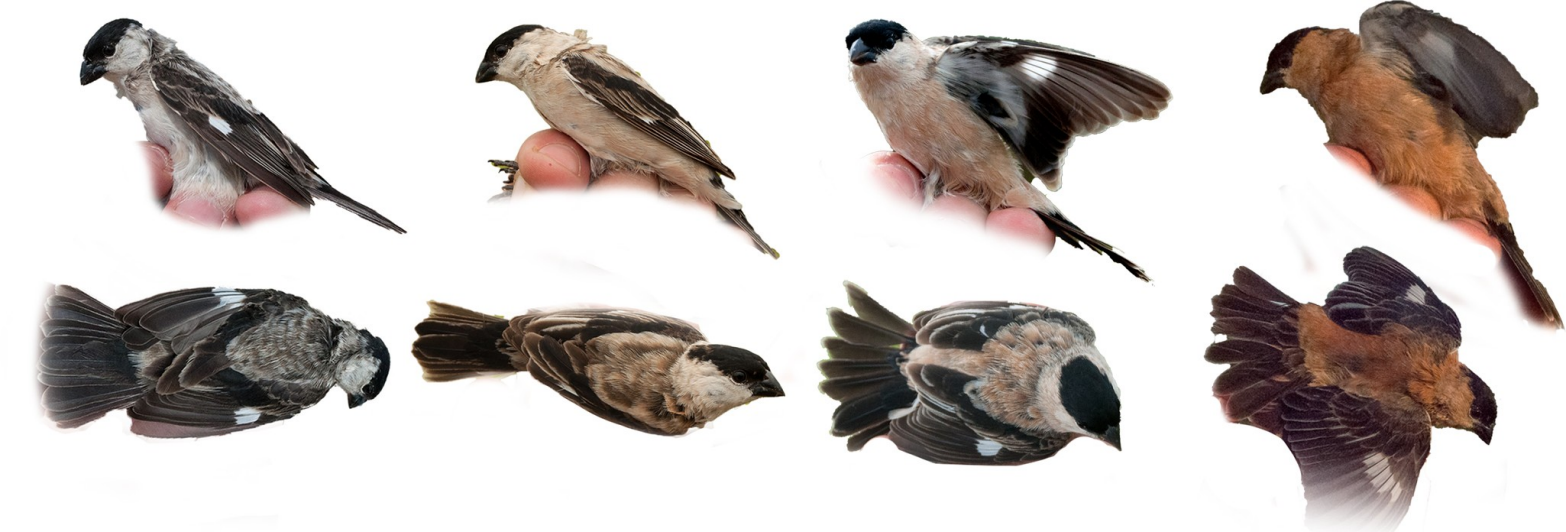
Pictures evidencing ventral and dorsal plumage scores for *Sporophila pileata*, *S*. *bouvreuil*, and intermediates. In the upper images, from left to right, the ventral color of a typical *S*. *pileata* (score 1); intermediate (score 2); intermediate (score 3), and a typical *S*. *bouvreuil*, with intense reddish-brown underparts (score 4). In the lower images, from left to right, the heavily streaked dorsal patterns of a typical *S*. *pileata* (score 1); intermediate (score 2); intermediate (score 3), and a typical *S*. *bouvreuil*, with a totally clean upperparts (score 4).

At each site, adult birds (males and females) were captured using mist nets of 12 X 2.5 m placed in the territories defended by males, and playback trials of their songs were used to increase capture rate. Because from mid-October to early November other capuchino species, such as *S*. *hypoxantha* and *S*. *melanogaster*, as well as potential individuals of *S*. *pileata* that belong to southern reproductive populations pass by São Paulo state during their migrations to the breeding areas of southern Brazil, only animals that were observed in our study sites throughout the breeding season (December/January) were considered in our analyses. As a result, it is unlikely that we have sampled individuals from other reproductive populations. Once captured, individuals were banded with a metal ring from CEMAVE/ICMBio, and with a combination of 1–3 PVC colored rings for posterior identification. Adult males had their ventral and dorsal parts photographed and they were scored from 1 to 4 based on their coloration. For the ventral parts, score (1) represented the typical *S*. *pileata*, with light-gray underparts coloration; (2) intermediate, with traits of gray and beige colorations; (3) intermediate, with traits of gray and reddish-brown, and (4) the typical *S*. *bouvreuil*, with intense reddish-brown lower parts. For the upperparts, (1) represented the typical *S*. *pileata*, with heavily streaked dark-gray dorsal region; (2) intermediate with predominantly streaked dorsal region; (3) intermediate, with predominantly clean dorsal region, and (4) the typical *S*. *bouvreuil*, with totally clean dorsal region (Fig 2). Individuals were considered phenotypically parental when they received the scores 1 or 4 for both ventral and dorsal characteristics, and animals that received scores 2 or 3 in at least one of the characteristics were hereafter considered as ''intermediates'' [see also 25, 26].

Afterwards, a blood sample of 5 μl was obtained from each animal by cutting the tip of a nail. Blood was mixed with approximately 10μl of 5M EDTA buffer, and added to a 1.5 ml tube containing 100% ethanol, and when in laboratory the samples were stored in freezer at -20°C. Genomic DNA was extracted using the kit ReliaPrep (TM) Blood DNA Miniprep System, PROMEGA, following the manufacturer protocol.

For paternity tests (see bellow), in SBO we sampled not only as many reproductive pairs as possible, but also their offspring. Nests were searched by checking the defended territories and by following parental individuals when they were carrying materials for nest construction or when they were feeding the nestlings [27]. Then, nests were monitored so that young could be captured with mist nets just after fledging, while they were still close to the nests and flying weakly. After marking and sampling, focal observations of post-fledging parental care were also performed to confirm social paternity of the offspring.

Field work and laboratorial procedures were in compliance with the Brazilian legislation. Birds capture and blood sampling were approved by Ministério do Meio Ambiente, Instituto Chico Mendes de Conservação da Biodiversidade, SISBIO/ICMBio (permit #21404–15), and by the Ethical Committee for the Use of Animals (CEUA: permit # 9257170616) from Universidade Federal de São Carlos, UFSCar. The Centro Nacional de Pesquisa e Conservação de Aves Silvestres (CEMAVE) provided the metal rings and permitted their use (permit #4022/6). Field work at the conservation unit EEI was authorized by the ConselhoTécnico Científico (COTEC: permit #260108-006638/2015), Instituto Florestal do Estado de São Paulo, IF. At SBO our work was authorized by the company owning the farm (Nova Amaralina S.A. Propriedades Agrícolas), and at SM permissions were obtained from the owners of a set of small properties.

### mtDNA analyses

We first constructed a phylogeny to recuperate the split between *S*. *pileata* and *S*. *bouvreuil*, from which haplotypes diagnostic of each taxa could be drawn. To achieve this purpose we PCR-amplified 631bp of the mitochondrial gene Cytochrome Oxidase I (COI; barcode), using the primers COIBirdF1 (TTCTCCAACCACAAAGACATTGGCAC) and COIbirdR2 (ACGTGGGAGATAATTCCAAATCCTGG) [28]. The COI was chosen because variable sites present in this gene were responsible for the identification of the Copper-seedeater as the sister taxa of the southern capuchinos in the study of [13]. PCR reactions were conducted in an Eppendorf MasterCycler Gradient thermocycler, in a 25 μl volume, containing 100 ng of DNA, 150 μM of each dNTP, 6.6 μl of the amplification buffer (200 mM Tris- HCl, pH 8.4 and 500 mM KCl; Promega), 0.4 μM of each primer, 2.5 μl of BSA (25 μg / ml) and 1 U Taq-Polymerase (Promega). The thermal cycler was programmed to an initial denaturing step of 5 min at 94°C, followed by 30 cycles of 94°C (30 s), 52.2°C (30 s), and 72°C (40 s), with a final extension of 72°C (10 min). PCR products were purified using the Wizard SV Gel and PCR Clean-Up System, Promega, and were sequenced on an ABI 3730 automated sequencer using the BigDye^®^ Terminator v3.1 Cycle Sequencing Kit.

For the phylogetic analysis, in addition to all of the individuals captured in our three study areas, we added 10 sequences of *S*. *pileata* (GU070595, GU070599, GU070598, GU070597, GU070596, GU070594, KF316357, KF316356, KF316354, KF316355), and 15 samples of *S*. *bouvreuil* (KF316360, KF316362, KF316363, KF316364, KF316365, KF316366, KF316367, KF316368, KF316369, KF316370, KF316371, KF316372, KF316373, KF316374, KF316375) that were used in the study of [13], all available from GenBank. All of these GenBank sequences were of adult males with typical plumages of each taxa, and were deposited in the ornithological collection of Museu de Zoologia da Universidade de São Paulo—MZUSP; these voucher specimens permitted us to check their color patterns again. Furthermore, one sequence of *S*. *minuta* DQ434137 was used as an outgroup [see 13]. One sample of each haplotype identified here for the first time was deposited in GenBank under accession numbers MN114257–MN114276. Sequences were aligned automatically by the CLUSTAL W procedure [29] implemented in Mega 7 [30], and we identified the haplotypes and calculated haplotype and nucleotide diversities, as well as the nucleotide composition using DNAsp 5.10.1 [31]. The Bayesian information criterion (BIC) implemented in the software PartitionFinder2 [32] was used to select the most appropriate nucleotide substitution model for each partition. All datasets were partitioned into first, second and third codon positions and the appropriate nucleotide substitution model was F81 [33] for the first, HKY+I [34] for the second, and K80+I [35] for the third codon position. The phylogeny was constructed using the Bayesian procedure of MrBayes 3.2.1 [36]. The analysis consisted of two independent runs of 20,000,000 generations, with one cold and three heated chains each, sampling trees every 1000 generations, and we adopted default parameter settings. We verified the convergence of the runs through the Potential Scale Reduction Factor (PSRF), which should approach 1.0 as runs converge [37]. For comparative purposes, we also generated a haplotype network using the median-joining method of the software Popart (http://popart.otago.ac.nz) [38].

### Paternity tests

For paternity tests, we used 17 microsatellite loci isolated from the *S*. *maximiliani*, being 14 described in [39]: Sma1, Sma4, Sma5, Sma6, Sma7, Sma8, Sma11, Sma13, Sma18, Sma21, Sma22, Sma25, Sma29, and Sma31, and three loci: Sma 20 ((AT)_21_; F: GGAACCTCTGCTGCTTG, R: TGCCTTCCTTAGCAGACTC), Sma 32 ((AC)_12_; F: GCCAGCTGAAATCCATAGGC, R: CTCTCCTGTGCTCCTTCCAG) and Sma 33 ((AT)_12_; F: GCTGCTTGAAATTCTCGTGC, R: CCTAAAGCTGGAAGTGTGG), also isolated from *S*. *maximiliani*, are presented here for the first time. Loci isolation procedures and PCR conditions are described in [39], and primer sequences, repeat motifs, and annealing temperatures optimized for *S*. *pileata* and *S*. *bouvreuil* by gradient PCR are presented in S1 Table. Primers were labeled with FAMN or HEX fluorescent dyes and amplification products were scored on an automated sequencer (ABI 3730) for genotyping. Allele sizes were analyzed using the software GENEMARKER 2.4.0 (Softgenetics). We estimated the observed (*H*_O_) and expected (*H*_E_) heterozygosities, probability of heterozygosity deficit, and the probability of linkage disequilibrium using the software GENEPOP 4.0 [40]. Evidence for null alleles, allelic dropout, and scoring errors due to stuttering were tested using Micro-Checker [41]. The accuracy of the set of loci to perform parentage analyses was assessed by the average probability that two random individuals in the population could present identical allelic composition (the probability of identity), and by estimating the probability that the set of loci could not exclude a pair of candidate unrelated parents from a random offspring, with the software Cervus 3.0 [42]. For the above estimates, only adult birds from SBO were used.

We considered Extra-Pair Paternity (EPP) when at least one young in a brood was not sired by at least one of its social parents [43], and we identified EPP by direct observation of allele inheritance [44, 45], and by estimating the maximum likelihood values of relatedness (r) provided by ML-Relate [46]. With the direct observation method, EPP resulted when allelic mismatching occurred in at least two loci, as mismatching at only one locus can be potentially caused by mutations or null alleles [47, 48]. With ML-Relate, EPP was considered whenever relatedness levels between social parents and offspring were other than “Parent-offspring”. As here we aim to confirm that couples with incongruent mtDNA haplotypes can sire offspring, parent-offspring relationship was confirmed only when both methods failed to indicate EPP.

## Results

### Adult birds sampling and plumage characterization

**Fig 3:**
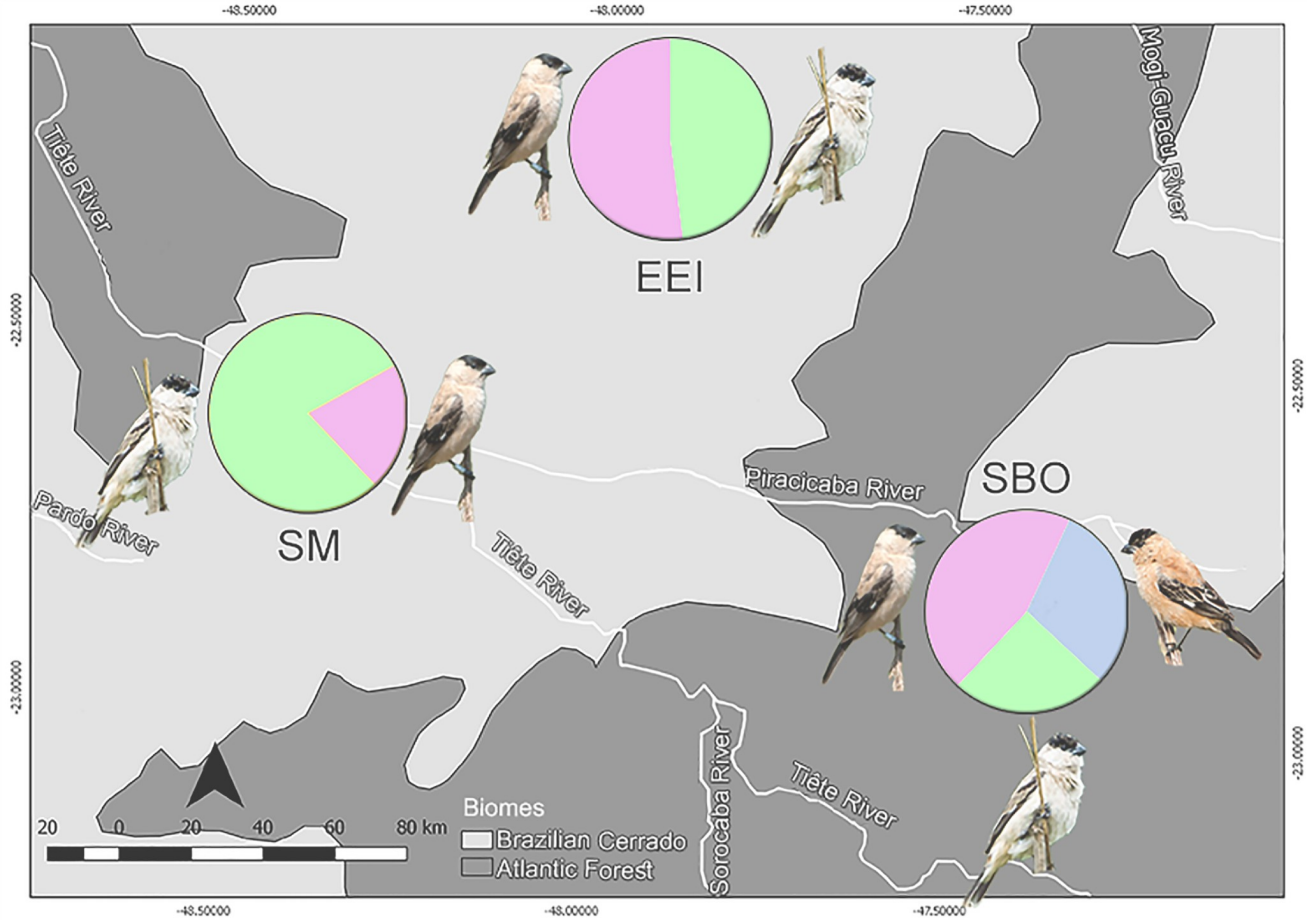
Proportions of males with different nuptial plumage patterns in three breeding areas from São Paulo state, Southeastern Brazil. Colors represent the typical *S*. *pileata* (green), the typical *S*. *bouvreuil* (blue), and intermediate (pink) patterns.

During two breeding seasons (2016/2017 and 2017/2018) we captured 91 adult birds in their territories across the three study areas. In SBO we sampled 20 adult males and 13 females. Among the males, five presented the typical phenotype of *S*. *pileata*, seven of *S*. *bouvreuil*, and eight had intermediate plumage patterns. In EEI we captured 33 males and two females, being 18 males with the *S*. *pileata* phenotype and 15 with intermediate plumage. In SM we captured 19 males and four females, being 15 males with the *S*. *pileata* phenotype and four with the intermediate phenotype. No individuals with the typical *S*. *bouvreuil* phenotype were registered in EEI and in SM (Fig 3). During the field work, we confirmed that these were the only species of capuchinos present in our study areas throughout the reproductive season.

### mtDNA analyses and maternal ancestry

The mtDNA PCR-reactions resulted in high-quality sequences of the mitochondrial gene COI for 79 individuals (32 from SBO, 26 from EEI, and 21 from SM). Including the GenBank sequences, our alignment evidenced 43 polymorphic sites, of which 16 were singleton variable sites, and 27 were parsimoniously informative sites. Haplotype diversity, nucleotide diversity, and nucleotide compositions are presented in Table 1. We are certain that the amplification products were of mitochondrial origin because we obtained single PCR products per individual; double-peaks were never observed, and our sequences were similar to those deposited in GenBank for closely related taxa.

**Table 1:** Characterization of the COI amplification products obtained from 104 *S*. *pileata*/*bouvreuil* samples. Numbers presented for variable sites represent the nucleotide positions homologous to the complete COI sequencing of *S*. *bouvreuil* (KF316362) [13].

Numbers of sequences	104
Numbers of Polymorphic sites	43
Singleton variable sites	220; 265; 280; 315; 352; 370; 373; 379; 394; 409; 412; 469; 512; 577; 634; 643
Parsimony informative variable sites	119; 127; 163; 268; 295; 316; 325; 331; 337; 340; 344; 355; 364; 367; 400; 404; 430; 496; 499; 520; 523; 535; 538; 541; 635; 649; 667
Haplotype (gene) diversity	0.9
Nucleotide diversity	0.0089
Nucleotide compositions	T 25.2%
	C 32.4%
	A 26.3%
	G 16.0%

**Fig 4:**
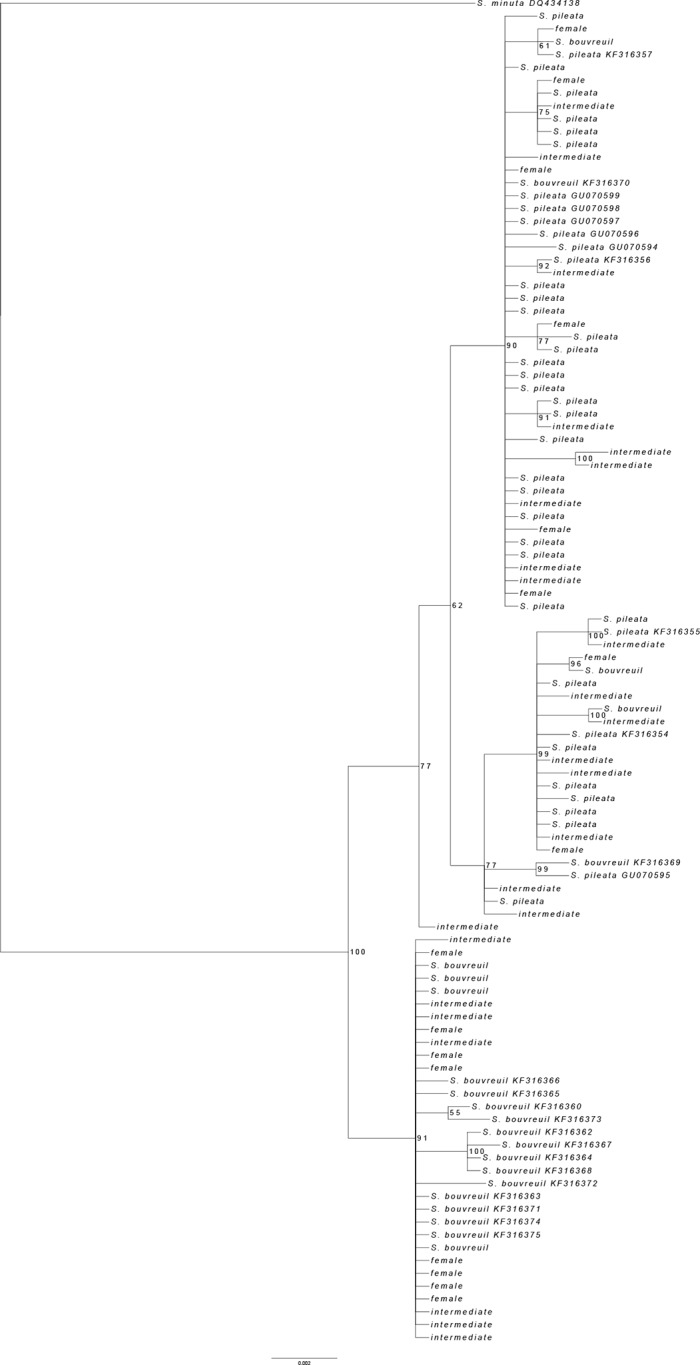
Bayesian phylogenetic tree based on COI haplotypes. The males are identified according to their phenotypes, i.e. typical *S*. *pileata* plumage; typical *S*. *bouvreuil* plumage, and intermediate color pattern (intermediate). Sequences previously available in GENBANK are identified with their voucher codes. Numbers represent Bayesian posterior probabilities.

The Bayesian maximum credibility tree revealed two distinct clades with high posterior probability support (Fig 4). The homologous sequences deposited in GENBANK identified as belonging to *S*. *pileata* and *S*. *bouvreuil* [13] remained within their respective clades, reinforcing the potential of this mtDNA region to identify these two species (Fig 4). The only exceptions were the sequences KF316369 and KF316370, for which mtDNA and morphology were incongruent, likely due to hybridization (see Discussion bellow).

**Fig 5:**
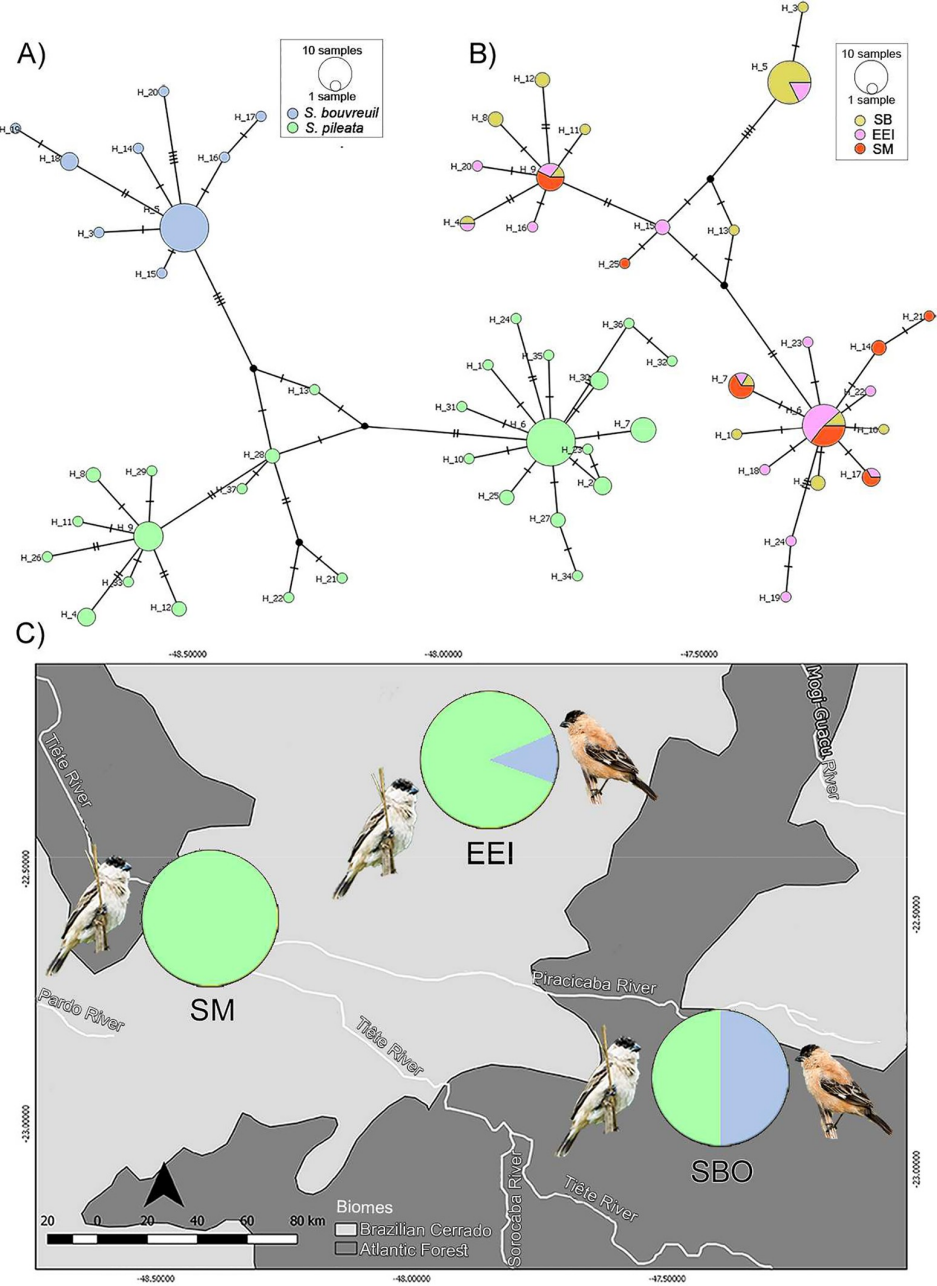
Median-joining networks and mtDNA haplotype frequencies across study areas. In the networks, circles represent distinct haplotypes and circle sizes are proportional to haplotype frequencies. A) Types and frequencies of haplotypes across the two different clades identified in the Bayesian phylogeny, including animals from GenBank; B) Types and frequencies of the different haplotypes considering only individuals sampled by us across the three study areas, and C) Image depicting the frequencies of haplotypes of each species in the three study sites. The haplotypes of the five individuals with *bouvreuil* morphology recovered in the *pileata* clade (including two individuals from GenBank) were here considered as haplotypes of *S*. *pileata*.

The median joining network revealed three main haplogroups, being one with the representatives of *S*. *bouvreuil* clade and two haplogroups representing the *S*. *pileata* clade (Fig 5A and 5B). Considering our samples and those from GenBank together, we found a total of 37 different haplotypes, with nine corresponding to *S*. *bouvreuil* and 28 to *S*. *pileata* groups (Fig 5A). Considering only the animals sampled by us, we identified 25 haplotypes across the three study areas, with 23 belonging to the *S*. *pileata* clade, and only two to the *S*. *bouvreuil* group (Fig 5B). Haplotypes of the *pileata* and *bouvreuil* clades were present in similar proportions among the 32 sequenced individuals from SBO, being 16 of the *pileata* and 16 of the *bouvreuil* clades. Among the 26 individuals sequenced from EEI, only 3 (11.5%) had haplotypes of the *bouvreuil* group, and among the 21 individuals from SM *bouvreuil* haplotypes were not found (Fig 5C). In Table 2 we present the combinations of coloration scores and mtDNA origin for the males in nuptial plumage captured in the three study areas, for which good mtDNA sequences were obtained. As ventral and dorsal scores always matched for the intermediates, these characteristics were not treated separately in the table. Although males classified in score 2 were closer to *pileata*, and those classified in score 3 were closer to *bouvreuil* parental individuals in morphology, haplotypes of *pileata* and *bouvreuil* were present in similar proportions within both of these morphological categories (29% of *bouvreuil* haplotypes in score 2, and 30% of *bouvreuil* haplotypes in score 3) (Table 2), suggesting that the combination of color patterns and mtDNA haplotypes is not sufficient to identify hybrid categories, i.e. F1 versus backcrosses.

**Table 2:** Numbers of males in nuptial plumage characterized by their coloration scores and mtDNA origin. Individuals with *Sporophila pileata* plumage phenotype received score 1, and individuals with *S*. *bouvreuil* plumage phenotype received score 4. Results are presented separately for each study area: Santa Bárbara d’Oeste (SBO), Estação Ecológica de Itirapina (EEI), and São Manuel (SM).

Study area	mtDNA origin	Coloration scores			
		Score 1	Score 2	Score 3	Score 4
SBO	*S*. *pileata*	5	2	2	3
	*S*. *bouvreuil*	–	2	2	4
EEI	*S*. *pileata*	12	5	4	–
	*S*. *bouvreuil*	–	2	1	–
SM	*S*. *pileata*	15	3	1	–
	*S*. *bouvreuil*	–	–	–	–

### Microsatellite analyses and paternity tests

Of the 17 cross-amplified microsatellite loci, six did not amplify satisfactorily, and three (Sma7, Sma20, and Sma33) were linked or presented evidence for null alleles, and were not used in subsequent analyses. The eight remaining loci presented from 5 to 20 alleles each, averaging 10.2 ± 5.63 alleles. *H*_O_ and *H*_E_ varied from 0.15 to 0.25 and from 0.11 to 0.25, and averaged 0.2 ± 0.04 and 0.18 ± 0.05, respectively (S1 Table). Significant deficit of heterozygotes was observed only for loci Sma5. The probability of identity of two random individuals in the population was < 0.000%, and the probability that the set of loci could not exclude an unrelated pair of parents was 0.008%.

In SBO we found 24 nests, being 10 in 2016/2017 and 14 in 2017/2018 breeding seasons. Of these nests, 11 were depredated during incubation or nestling stages, so that we could mark and sample parental and fledglings for 11 of these nests, totaling 13 fledglings from nine couples, and from one family for which only the adult male was sampled. Notably, one of our study couples produced three clutches during the two breeding seasons. In four of these pairs males and females showed mtDNA haplotypes belonging to different clades; three pairs were of representatives of the *bouvreuil*, and two pairs were of representatives of the *pileata* clades. For two of the four pairs of individuals with incongruent mtDNA haplotypes paternity tests have confirmed that the social parents have sired the young, while EPP was observed in the other two pairs. EPP was not identified among the three families for which parents had *bouvreuil* haplotypes, but EPP was confirmed for two nests of the two pairs of individuals with *pileata* mtDNA. Notably, an extra-pair young for which social parents had incongruent haplotypes (male with *pileata*, and female with *bouvreuil* haplotype) had its paternity attributed to the male of the neighboring territory, that had a *bouvreuil* haplotype. All of the cases of parental/offspring mismatch resulted from father exclusion. Genotypings used in paternity analyses and allelic mismatches are indicated in S2 Table, and relatedness levels estimates for the pairs of individuals obtained with ML-Relate are presented in S3 Table. Together, this suggests that individuals with incongruent mtDNA can interbreed and produce offspring that were viable to the fledging age.

Among the four males for which offspring siring was confirmed by paternity tests, one had *pileata* phenotype and mtDNA, and three had intermediate plumage patterns and *bouvreuil* mtDNA. Independently of nest detection, 12 territorial pairs for which both males and females were sampled and marked presented the following haplotypic combinations: male/*bouvreuil* and female/*bouvreuil* (n = 3); male/*bouvreuil* and female/*pileata* (n = 2); male/*pileata* and female/*bouvreuil* (n = 4); male/*pileata* and female/*pileata* (n = 3).

## Discussion

Addressing hybridization in recently diverged taxa is not straightforward, being the discrimination between incomplete lineage sorting and hybridization the main challenge [1–4, 13]. Here, however, data give support to the idea that *S*. *pileata* and *S*. *bouvreuil* can hybridize in nature. The main evidence include: i) the widely disjunct distribution of the two species with records of individuals with intermediate plumage patter concentrated near the contact zone [see 20, 21]; ii) the similar proportion of haplotypes belonging to *pileata* and *bouvreuil* clades only in the area embedded within the contact zone, the only of our three areas in which we found the two typical parental plumage patterns together; iii) the presence of males with intermediate plumage in both of the well-supported clades; iv) the decreasing frequencies of males with intermediate plumage and of mtDNA haplotypes that are exclusive of the *bouvreuil* clade in the direction of the core area of *pileata*, and v) the fact that intermediate plumage patterns occur frequently in nature only in this pair of southern capuchinos, differently from the other congeners that do reproduce in sympatry.

In the study of [13], two of 19 individuals with typical adult male plumage of *S*. *bouvreuil* presented mtDNA sequences of the genes COI, Cyt b, and Control Region that placed them within the southern capuchinos clade, rather than in the group of *bouvreuil*. These authors have attributed this finding to incomplete lineage sorting, which could lead to the sharing of some mtDNA haplotypes between species, or to hybridization, which could not be confirmed without population level analyses. GenBank sequences from these two individuals were included in our phylogenetic reconstruction, and together with three other typical *bouvreuil* sampled by us, they were again placed in the *pileata* clade. We assessed the voucher specimens of *S*. *bouvreuil* deposited at MZUSP to check their origins, and they were collected during migration in Northern Mato Grosso state, northern Brazil, in a region that is at least 900 km far from the northern distribution limits of *S*. *pileata*. Although this is a wintering area of individuals with unknown reproductive region, these groups were composed exclusively by individuals with the typical *S*. *bouvreuil* plumage. It is difficult to assert that *bouvreuil* do not share ancestral polymorphisms with the other congeners, but this species has a set of closely related ancestral haplotypes, to which the majority of males with the typical *bouvreuil* plumage belong, and that is responsible for the discrimination of *bouvreuil* from the other congeners [13]. Our analyses added new elements to this group of haplotypes and our inferences about the decreasing frequencies of *bouvreuil* haplotypes away from the contact zone is based on the haplotypes of this ancestral clade, i.e. haplotypes that represent exclusively *S*. *bouvreuil*. Together with the above listed body of evidence, it is parsimonious to consider that at least part of the males, from our study areas and also from GenBank, with *bouvreuil* plumage misassigned to the *pileata* clade (5 of 22; 23%) are highly backcrossed hybrids. Then, even if a certain level of incomplete lineage sorting exists, it is in a smaller scale and it does not preclude our main conclusion that hybridization occurs between *S*. *bouvreuil* and *S*. *pileata*. Our data also suggest that the pinkish-gray plumage pattern previously thought to be within the range of variation of *S*. *pileata* [21], may result from hybridization.

The confirmation of hybridizations by paternity tests, however, rely on the assumption that incomplete lineage sorting does not exist between the analyzed taxa, so that the main conclusion that can be drawned from this analysis is that individuals belonging to the two divergent haplogroups can interbred and produce offspring that are viable to fledging age. Further, if our conclusion that males with intermediate plumage are hybrids is true, the paternity confirmation for four of these males suggest that at least some of the hybrid categories are fertile.

Notably, two clearly distinct haplotypic groups were found within *S*. *pileata*, even with the exclusion of the samples from GenBank, which could result from the i) introgression from capuchinos species other than the *S*. *bouvreuil*, ii) occurrence of cryptic taxa in sympatry, or iii) secondary contact and reverse speciation of previously diverging lineages [49, 50]. As these are the only capuchino species reproducing in our study areas and these are the only southern capuchinos that do reproduce northern to São Paulo state, it is unlikely that contemporary introgression from other species have occurred in our sampling sites. *Sporophila pileata* may come into contact with breeding populations of other capuchinos in the southern parts of its reproductive distribution; however, there are no morphological cues for the presence of hybrids between *S*. *pileata* and other capuchinos like the ones observed here, reducing the evidence for the hypothesis of contemporary gene flow to explain the presence of two haplotypic groups within the *S*. *pileata* clade. The lack of complete reproductive isolation mechanisms between the diagnosable *S*. *pileata* and *S*. *bouvreuil* is *per se* evidence against the existence of cryptic and reproductively isolated lineages of *S*. *pileata* living in sympatry in our study areas. Then, based on the available information, the secondary contact and posterior homogenization of previously diverging lineages seems the most plausible hypothesis to explain the two distinct haplotypic groups within *S*. *pileata*. This theory has already been proposed by [10] as one of the mechanisms involved in the radiation of the southern capuchinos, and it may be one of the factors explaining the high COI haplotypic diversity within many of these species. However, these conclusions must be viewed with caution because the reproductive distributions of many of the southern capuchinos are still not fully understood and population samplings in contact zones are limited. Further, we are uncertain if these haplogroups are general to the entire southern capuchino clade.

The phylogenetic distinctiveness between *S*. *bouvreuil* and *S*. *pileata*, with clades corresponding to geographic areas, is characteristic of hybridization after secondary contact [1], and as our study was carried out in a short geographic scale we are unaware about how far hybridization could go into the distribution of the parental species. The higher frequency of males with intermediate plumage patterns than mtDNA haplotypes in Itirapina and in St. Bárbara d’Oeste suggest that introgression in nuclear markers can be higher than in mtDNA, a pattern that is well documented in the literature [see 51]. Then, future analyses involving more populations and more nuclear markers will contribute to elucidate the dynamic of the hybrid zone, revealing, for instance, if potential introgressions have been asymmetric or even if there is a tendency for reverse speciation [52, 26]. *Sporophila pileata* has been observed nesting in both well-preserved (Itirapina) and in degraded marshlands (São Manuel), while reproductive populations in which individuals with the typical *bouvreuil* phenotype predominates are still undescribed to science. If habitat disturbance has implied in movements of a possible hybrid zone [26] is another important and still open question.

The evidence for hybridization mean that the reproductive isolation mechanisms based on song and color pattern recognition present in the capuchinos that do reproduce in sympatry in southern areas have reduced importance for mate choices between *S*. *pileata* and *S*. *bouvreuil*. The patterns of decreasing frequencies of *bouvreuil* haplotypes and morphology into the core area of *pileata* provides more support to a scenario of hybridization in the contact zone, and maintenance of both taxa in their core areas by ecological, rather than by sexual selection mechanisms [53]. In the scenario proposed by [17] capuchinos that experienced color divergence during isolation periods in Plesistocenic refugia and developed strong sexual selection could maintain their independent evolutionary trajectories in sympatry, even with very little niche divergence. Here we expand this theory by showing that during the capuchino’s radiation, a pair of species with divergent geographic distribution and less color divergence [17] can hybridize in the contact zone. In addition to the taxonomic issues, recently diverged species that come into early secondary contact are of relevance because they provide insights into the processes involved in the first diversification steps. Studies on reproductive isolation mechanisms in well-diverged species have long been an important focus of evolutionary science, but studies involving recently diverged organisms are still less represented, and cases in which crossbreedings are maintained are of special interest [54–56]. Then, identifying the different hybrid classes with a higher number of genetic markers and analyzing their survival and reproductive success within a potential hybrid zone and in the introgressed areas are in the scope of our future works. In summary, our findings corroborated the subdivision of *S*. *bouvreuil* and *S*. *pileata* as distinct taxa, and elucidate the nature of the variations that have long confounded taxonomists. As the capuchinos represent one of the most recent and explosive avian radiations ever documented [10], the uncover of hybridization open new investigation perspectives that could contribute to the understanding of the radiation of this group.

## Supporting information

S1 Table Microsatellite loci characterization. Repeat motifs, primer sequences, PCR annealing temperatures (T_A_), numbers of alleles (N_A_), allele size ranges in base pairs (bp), observed (*H*_O_) and expected (*H*_E_) heterozygosities, probability of heterozygote deficits (*P*), probabilities that two random individuals in the population could present identical allelic composition (I), and the probability that the loci would not exclude a pair of candidate unrelated parents (PP) for eight microsatellite loci used for paternity tests in *S*. *pileata*/*bouvreuil*.
(DOCX)

S2 Table Raw microsatellite genotypic data for families of *Sporophila pileata*/*bouvreuil*. Discordant alleles among nestlings and social parents are marked in bold. Lines of young resulted from EPP are highlighted in gray.
(DOCX)

S3 Table Matrix of parent/offspring relationships obtained with ML-relate. M, F, and O indicate family’s male, female, and offspring, respectively. Symbols within the cells represent the highest likelihoods of each pairs of individuals to belong to one of the following categories: U = Unrelated; HS = Half Sibs; FS = Full Sibs, and PO = Parent/Offspring.
(DOCX)
