# Supplementary material for: Hybridization between two recently diverged Neotropical passerines: The Pearly-bellied Seedeater Sporophila pileata, and the Copper Seedeater S. bouvreuil (Aves, Passeriformes, Thraupidae)
Source: PLoS One. 2020 Mar 27;15(3):e0229714. doi: 10.1371/journal.pone.0229714 (PMC7100935; doi:10.1371/journal.pone.0229714)
Supplement: S1 Table — Repeat motifs, primer sequences, PCR annealing temperatures (TA), numbers of alleles (NA), allele size ranges in base pairs (bp), observed (HO) and expected (HE) heterozygosities, probability of heterozygote deficits (P), probabilities that two random individuals in the population could present identical allelic composition (I), and the probability that the loci would not exclude a pair of candidate unrelated parents (PP) for eight microsatellite loci used for paternity tests in S. pileata/bouvreuil. (DOCX) [file pone.0229714.s001.docx]

**S1 Table. Microsatellite loci characterization.** Repeat motifs, primer sequences, PCR annealing temperatures (T_A_), numbers of alleles (N_A_), allele size ranges in base pairs (bp), observed (*H*_O_) and expected (*H*_E_) heterozygosities, probability of heterozygote deficits (*P*), probabilities that two random individuals in the population could present identical allelic composition (I), and the probability that the loci would not exclude a pair of candidate unrelated parents (PP) for eight microsatellite loci used for paternity tests in *S. pileata*/*bouvreuil*.

| ***Locus*** | ***Motif*** | **Primer sequences (5’ – 3’)** | **T_A_ (°C)** | **N_A_** | **Allele range (bp)** | ***H*_E_** | ***H*_O_** | ***P**** | ***I*** | ***PP*** |
| --- | --- | --- | --- | --- | --- | --- | --- | --- | --- | --- |
| Sma5 | (AAT)_16_ | F: GTCCTTGCATGTGACAGTGG | 64.7 | 10 | 331-370 | 0.23 | 0.11 | 0.006 | 0.236 | 0.499 |
|  |  | R: CAGTCAGTGTGCCATGAACC |  |  |  |  |  |  |  |  |
| Sma11 | (AC)_15_ | F: TGAAATTGAACCATCCAGTGCC | 64.7 | 20 | 217-261 | 0.25 | 0.25 | 0.438 | 0.451 | 0.157 |
|  |  | R: ACCATAGTTCCATTGCTTCCAG |  |  |  |  |  |  |  |  |
| Sma21 | (AT)_12_ | F: GAGCAGGTACAAGGACTC | 64.5 | 5 | 255-287 | 0.15 | 0.12 | 0.056 | 0.016 | 0.056 |
|  |  | R: GATCTCCACTTCCTCCAGC |  |  |  |  |  |  |  |  |
| Sma22 | (AG)_13_ | F: GCATCTTCTGTACTGAGAGC | 63.3 | 6 | 402-418 | 0.18 | 0.15 | 0.056 | 0.220 | 0.464 |
|  |  | R: AACCTCAACAGACACGGAAC |  |  |  |  |  |  |  |  |
| Sma25 | (AC)_14_ | F: CTGTAGAAGAACTGCCAG | 61.4 | 18 | 216-278 | 0.24 | 0.24 | 0.307 | 0.129 | 0.312 |
|  |  | R: ACACTCTCACACAGCTTTGC |  |  |  |  |  |  |  |  |
| Sma29 | (AC)_14_ | F: CAAACAGGGCAAAGGGTGAC | 64.5 | 7 | 171-195 | 0.15 | 0.17 | 0.825 | 0.088 | 0.252 |
|  |  | R: TCAGCCTCGAGTTAACAC |  |  |  |  |  |  |  |  |
| Sma31 | (AC)_14_ | F: AGAAGTAGTCCCTCTAGC | 61.4 | 8 | 161-181 | 0.18 | 0.18 | 0.431 | 0.010 | 0.037 |
|  |  | R: TTGTCTGACTGGCTTGTAG |  |  |  |  |  |  |  |  |
| Sma32 | (AC)_12_ | F: GCCAGCTGAAATCCATAGGC | 64.5 | 8 | 355-371 | 0.21 | 0.22 | 0.769 | 0.035 | 0.117 |
|  |  | R: CTCTCCTGTGCTCCTTCCAG |  |  |  |  |  |  |  |  |

* Corrected *P* values after Bonferroni correction is 0.006.
