# Supplementary material for: Hybridization between two recently diverged Neotropical passerines: The Pearly-bellied Seedeater Sporophila pileata, and the Copper Seedeater S. bouvreuil (Aves, Passeriformes, Thraupidae)
Source: PLoS One. 2020 Mar 27;15(3):e0229714. doi: 10.1371/journal.pone.0229714 (PMC7100935; doi:10.1371/journal.pone.0229714)
Supplement: S2 Table — Discordant alleles among nestlings and social parents are marked in bold. Lines of young resulted from EPP are highlighted in gray. (DOCX) [file pone.0229714.s002.docx]

**S2 Table. Raw microsatellite genotypic data for families of *Sporophila pileata*/*bouvreuil*.** Discordant alleles among nestlings and social parents are marked in bold. Lines of young resulted from EPP are highlighted in gray.

| Individual | mtDNA | Loci | | | | | | | | | | | | | | | |
| --- | --- | --- | --- | --- | --- | --- | --- | --- | --- | --- | --- | --- | --- | --- | --- | --- | --- |
|  |  | Sma21a | Sma21b | Sma22a | Sma22b | Sma25a | Sma25b | Sma29a | Sma29b | Sma31a | Sma31b | Sma32a | Sma32b | Sma5a | Sma5b | Sma11a | Sma11b |
| M1 | *bouvreuil* | 255 | 255 | 406 | 406 | 232 | 238 | 179 | 185 | 167 | 169 | 363 | 365 | 337 | 337 | 249 | 253 |
| F1 | *bouvreuil* | 263 | 263 | 408 | 418 | 216 | 248 | 185 | 187 | 167 | 169 | 359 | 363 | 334 | 340 | 237 | 237 |
| O |  | 255 | 263 | 406 | 408 | 238 | 248 | 185 | 187 | 167 | 167 | 359 | 363 | 337 | 340 | **227** | **227** |
| O |  | 255 | 263 | 406 | 418 | 232 | 248 | 179 | 185 | 167 | 167 | 359 | 363 | 334 | **346** | 237 | 249 |
| M1 | *bouvreuil* | 255 | 255 | 406 | 406 | 232 | 238 | 179 | 185 | 167 | 169 | 363 | 365 | 337 | 337 | 249 | 253 |
| F1 | *bouvreuil* | 263 | 263 | 408 | 418 | 216 | 248 | 185 | 187 | 167 | 169 | 359 | 363 | 334 | 340 | 237 | 237 |
| O |  | 255 | 263 | 406 | 418 | 238 | 248 | 185 | 187 | 169 | 169 | 363 | 365 | 337 | 340 | 249 | 253 |
| O |  | 255 | 263 | 406 | 418 | 216 | 232 | 185 | 185 | 167 | 169 | 363 | 365 | 334 | 337 | 237 | 249 |
| M1 | *bouvreuil* | 255 | 255 | 406 | 406 | 232 | 238 | 179 | 185 | 167 | 169 | 363 | 365 | 337 | 337 | 249 | 253 |
| F1 | *bouvreuil* | 263 | 263 | 408 | 418 | 216 | 248 | 185 | 187 | 167 | 169 | 359 | 363 | 334 | 340 | 237 | 237 |
| O |  | 255 | 263 | 406 | 408 | 216 | 232 | 179 | 185 | 169 | 169 | 359 | 363 | 337 | 340 | 249 | 249 |
| M2 | *pileata* | 255 | 263 | 406 | 406 | 238 | 272 | 185 | 185 | 167 | 177 | 355 | 357 | 337 | 337 | 239 | 241 |
| F2 | *pileata* | 255 | 263 | 406 | 416 | 232 | 262 | 181 | 189 | 167 | 167 | 365 | 371 | 340 | 340 | 217 | 233 |
| O |  | 255 | **261** | 406 | 416 | 232 | **232** | 185 | 189 | 167 | 167 | **363** | 371 | **340** | 340 | 217 | **253** |
| O |  | 263 | 263 | 406 | 406 | 232 | **244** | 181 | 185 | 167 | 167 | **365** | 365 | 340 | **346** | 233 | **253** |
|  |  |  |  |  |  |  |  |  |  |  |  |  |  |  |  |  |  |
| M3 | *pileata* | 263 | 263 | 406 | 408 | 232 | 256 | 179 | 181 | 167 | 167 | 365 | 367 | 337 | 337 | 231 | 231 |
| F3 | *bouvreuil* | 263 | 263 | 406 | 408 | 232 | 258 | 185 | 185 | 167 | 167 | 363 | 365 | 337 | 337 | 227 | 241 |
| O |  | 263 | 263 | 406 | 408 | 232 | 232 | 185 | **185** | 167 | 167 | 363 | 365 | 337 |  | 227 | **243** |
|  |  |  |  |  |  |  |  |  |  |  |  |  |  |  |  |  |  |
| M4 | *pileata* | 287 | 287 | 408 | 408 | 234 | 242 | 179 | 185 | 165 | 167 | 359 | 363 | 352 | 352 | 231 | 243 |
| F4 | *bouvreuil* | 263 | 263 | 406 | 418 | 230 | 254 | 185 | 185 | 167 | 167 | 361 | 363 | 358 | 358 | 217 | 231 |
| O |  | **255** | 263 | **406** | 406 | 230 | **238** | 179 | 185 | 167 | 167 | 361 | **365** | **337** | **337** | 217 | **249** |
|  |  |  |  |  |  |  |  |  |  |  |  |  |  |  |  |  |  |
| M5 | *pileata* | 261 | 263 | 406 | 406 | 232 | 244 | 185 | 185 | 167 | 167 | 355 | 363 | 343 | 346 | 255 | 255 |
| F5 | *bouvreuil* | 255 | 263 | 408 | 408 | 234 | 234 | 179 | 185 | 167 | 171 | 359 | 365 | 337 | 343 | 225 | 253 |
| O |  | 255 | 261 | 406 | 408 | 232 | 234 | 185 | 185 | 167 | 167 | 355 | 365 | 343 | 343 | 225 | 255 |
| O |  | 263 | 263 | 406 | 408 | 232 | 234 | 185 | 185 | 167 | 171 | 355 | 365 | 346 | 346 | 225 | 255 |
|  |  |  |  |  |  |  |  |  |  |  |  |  |  |  |  |  |  |
| M6 | *bouvreuil* | 261 | 263 | 408 | 408 | 234 | 278 | 179 | 185 | 165 | 171 | 357 | 363 | 346 | 352 | 229 | 243 |
| F6 | *bouvreuil* | 263 | 263 | 408 | 408 | 234 | 234 | 179 | 185 | 167 | 177 | 359 | 365 | 337 | 337 | 227 | 229 |
| O |  | 263 | 263 | 408 | 408 | **258** | 278 | 179 | 185 | 167 | 171 | 363 | 365 | 337 | 346 | 227 | 229 |
|  |  |  |  |  |  |  |  |  |  |  |  |  |  |  |  |  |  |
| M7 | *pileata* | 263 | 263 | 402 | 406 | 234 | 238 | 185 | 185 | 169 | 175 | 355 | 359 | 346 | 346 | 241 | 245 |
| O | *pileata* | 263 | 263 | **408** | **408** | 234 | 258 | 179 | 185 | **167** | **167** | 363 | 365 | 337 | **340** | 227 | **243** |
|  |  |  |  |  |  |  |  |  |  |  |  |  |  |  |  |  |  |
| M8 | *bouvreuil* | 255 | 263 | 406 | 406 | 216 | 232 | 179 | 185 | 167 | 171 | 365 | 365 | 340 | 340 | 229 | 241 |
| F4 | *bouvreuil* | 263 | 263 | 406 | 418 | 230 | 254 | 185 | 185 | 167 | 167 | 361 | 363 | 358 | 358 | 217 | 231 |
| O |  | 255 | 263 | 406 | 418 | 216 | 254 | 179 | 185 | 167 | 167 | 363 | 365 | 340 | 358 | 229 | 231 |
|  |  |  |  |  |  |  |  |  |  |  |  |  |  |  |  |  |  |
| M9 | *bouvreuil* | 255 | 263 | 406 | 406 | 228 | 244 | 179 | 181 | 169 | 169 | 363 | 367 | 343 | 358 | 229 | 247 |
| F9 | *pileata* | 255 | 263 | 406 | 406 | 228 | 244 | 181 | 181 | 165 | 167 | 359 | 367 | 346 | 355 | 247 | 255 |
| O |  | 263 | 263 | 406 | **410** | 228 | **232** | 179 | 181 | 167 | **171** | 359 | **365** | 346 | **346** | **233** | 255 |
