# Supplementary material for: Hybridization between two recently diverged Neotropical passerines: The Pearly-bellied Seedeater Sporophila pileata, and the Copper Seedeater S. bouvreuil (Aves, Passeriformes, Thraupidae)
Source: PLoS One. 2020 Mar 27;15(3):e0229714. doi: 10.1371/journal.pone.0229714 (PMC7100935; doi:10.1371/journal.pone.0229714)
Supplement: S3 Table — M, F, and O indicate family’s male, female, and offspring, respectively. Symbols within the cells represent the highest likelihoods of each pairs of individuals to belong to one of the following categories: U = Unrelated; HS = Half Sibs; FS = Full Sibs, and PO = Parent/Offspring. (DOCX) [file pone.0229714.s003.docx]

**S3 Table. Matrix of parent/offspring relationships obtained with ML-relate.** M, F, and O indicate family's male, female, and offspring, respectively. Symbols within the cells represent the highest likelihoods of each pairs of individuals to belong to one of the following categories: U = Unrelated; HS= Half Sibs; FS = Full Sibs, and PO = Parent/Offspring.

|  | M1 | F1 | O | O | O | O | O | M2 | F2 | O | O | M3 | F3 | O | M4 | F4 | O | M5 | F5 | O | O | M6 | F6 | O | M7 | O | M8 | O | M9 | F9 | O |
| --- | --- | --- | --- | --- | --- | --- | --- | --- | --- | --- | --- | --- | --- | --- | --- | --- | --- | --- | --- | --- | --- | --- | --- | --- | --- | --- | --- | --- | --- | --- | --- |
| M1 | - |  |  |  |  |  |  |  |  |  |  |  |  |  |  |  |  |  |  |  |  |  |  |  |  |  |  |  |  |  |  |
| F1 | U | - |  |  |  |  |  |  |  |  |  |  |  |  |  |  |  |  |  |  |  |  |  |  |  |  |  |  |  |  |  |
| O | PO | HS | - |  |  |  |  |  |  |  |  |  |  |  |  |  |  |  |  |  |  |  |  |  |  |  |  |  |  |  |  |
| O | HS | PO | U | - |  |  |  |  |  |  |  |  |  |  |  |  |  |  |  |  |  |  |  |  |  |  |  |  |  |  |  |
| O | HS | HS | FS | U | - |  |  |  |  |  |  |  |  |  |  |  |  |  |  |  |  |  |  |  |  |  |  |  |  |  |  |
| O | PO | PO | U | FS | HS | - |  |  |  |  |  |  |  |  |  |  |  |  |  |  |  |  |  |  |  |  |  |  |  |  |  |
| O | PO | HS | U | HS | U | PO | - |  |  |  |  |  |  |  |  |  |  |  |  |  |  |  |  |  |  |  |  |  |  |  |  |
| M2 | HS | U | U | U | U | U | U | - |  |  |  |  |  |  |  |  |  |  |  |  |  |  |  |  |  |  |  |  |  |  |  |
| F2 | U | U | U | U | U | U | U | U | - |  |  |  |  |  |  |  |  |  |  |  |  |  |  |  |  |  |  |  |  |  |  |
| O | HS | U | HS | U | U | U | U | U | FS | - |  |  |  |  |  |  |  |  |  |  |  |  |  |  |  |  |  |  |  |  |  |
| O | U | U | U | U | U | U | U | U | PO | U | - |  |  |  |  |  |  |  |  |  |  |  |  |  |  |  |  |  |  |  |  |
| M3 | U | U | U | U | U | U | U | U | U | U | U | - |  |  |  |  |  |  |  |  |  |  |  |  |  |  |  |  |  |  |  |
| F3 | U | U | U | U | U | U | U | HS | U | U | U | U | - |  |  |  |  |  |  |  |  |  |  |  |  |  |  |  |  |  |  |
| O | U | U | U | U | U | HS | U | U | U | U | U | HS | FS | - |  |  |  |  |  |  |  |  |  |  |  |  |  |  |  |  |  |
| M4 | U | U | U | U | U | U | U | U | U | U | U | U | U | U | - |  |  |  |  |  |  |  |  |  |  |  |  |  |  |  |  |
| F4 | U | U | U | U | U | U | U | U | U | U | U | U | U | U | U | - |  |  |  |  |  |  |  |  |  |  |  |  |  |  |  |
| O | PO | U | U | U | U | U | U | HS | HS | U | U | U | U | U | U | HS | - |  |  |  |  |  |  |  |  |  |  |  |  |  |  |
| M5 | U | U | U | U | U | U | U | U | U | HS | HS | U | U | U | U | U | U | - |  |  |  |  |  |  |  |  |  |  |  |  |  |
| F5 | U | U | HS | U | U | U | U | U | U | U | U | U | U | U | U | U | U | U | - |  |  |  |  |  |  |  |  |  |  |  |  |
| O | U | U | U | U | U | U | U | U | U | U | U | U | U | U | U | U | U | PO | PO | - |  |  |  |  |  |  |  |  |  |  |  |
| O | U | U | U | U | U | U | U | U | U | U | U | U | U | U | U | U | U | PO | HS | FS | - |  |  |  |  |  |  |  |  |  |  |
| M6 | U | U | U | U | U | U | U | U | U | U | U | U | U | U | HS | U | U | U | U | U | HS | - |  |  |  |  |  |  |  |  |  |
| F6 | U | U | U | U | U | U | U | U | U | U | U | U | HS | HS | U | U | U | U | FS | U | U | U | - |  |  |  |  |  |  |  |  |
| O | U | U | U | U | U | U | U | U | U | U | U | U | PO | HS | U | U | U | U | U | U | HS | PO | FS | - |  |  |  |  |  |  |  |
| M7 | U | U | U | U | U | U | U | HS | U | U | U | U | U | U | U | U | U | U | U | U | HS | U | U | U | - |  |  |  |  |  |  |
| O | U | U | U | U | U | U | U | U | U | U | U | U | PO | FS | HS | U | U | U | HS | U | U | HS | PO | PO | U | - |  |  |  |  |  |
| M8 | U | U | U | U | U | U | U | U | U | U | U | U | HS | U | U | U | U | U | U | U | U | U | U | U | U | U | - |  |  |  |  |
| O | U | HS | U | U | U | HS | U | U | U | U | U | U | U | U | U | PO | U | U | U | U | U | U | U | U | U | U | PO | - |  |  |  |
| M9 | U | U | U | U | HS | U | U | U | U | U | U | U | U | U | U | U | U | U | U | U | U | U | U | U | U | U | U | HS | - |  |  |
| F9 | U | U | U | U | U | U | U | U | U | U | HS | U | U | U | U | U | U | HS | U | U | U | U | U | U | U | U | U | U | FS | - |  |
| O | U | U | U | U | U | U | U | U | U | U | HS | U | U | U | U | U | U | HS | U | U | FS | U | U | U | U | U | U | U | U | PO | - |
